# Effect of intravenous sedation on patients’ visual experience and vital signs during cataract surgery under topical anesthesia: A randomized controlled trial

**DOI:** 10.1016/j.aopr.2021.100006

**Published:** 2021-09-09

**Authors:** Rengaraj Venkatesh, Hemal Kenia, Sabyasachi Sengupta, Megha Gopalakrishna, Kah-Guan Au Eong

**Affiliations:** a Aravind Eye Hospital, Pondicherry, India; b Future Vision Eye Care and Research Center, Mumbai, India; c International Eye Cataract Retina Center, Mount Elizabeth Medical Center and Farrer Park Medical Center, Singapore

**Keywords:** Midazolam, Visual experience, Phacoemulsification, Cataract surgery, Sedation

## Abstract

**Purpose:**

Effect of intravenous sedation on patients’ visual experience and vital signs during cataract surgery under topical anesthesia: a randomized controlled trial.

**Design:**

Prospective, double masked, randomized controlled trial.

**Methods:**

150 eyes of 150 patients undergoing phacoemulsification and IOL implantation under topical anesthesia were randomized to receive either intravenous midazolam (0.015 ​mg/kg) or normal saline. The patients’ experience was evaluated using a questionnaire. Vital signs including blood pressure and heart rate were measured before, during and after surgery. Mean arterial pressure (MAP) was calculated.

**Results:**

Both groups were comparable except that fewer patients in the control group were pseudophakic in the fellow eye (25.3% vs. 41.3%). More patients in the control group perceived hand movements (p ​< ​0.01), surgeon/medical staff (p ​= ​0.04) and sudden increase in vision during surgery (p ​< ​0.01) compared to midazolam group. More control group patients experienced fear (p ​< ​0.001), pain (p ​= ​0.06) and unpleasant surgical experience (20.3% vs. 1.3%, p ​< ​0.001). They also experienced greater fluctuation in MAP (16.9 ​± ​7.9 vs.7.2 ​± ​5.3, p ​< ​0.001) and this was accentuated in hypertensives. After adjusting for age, gender, hypertension status and other eye lens status, multivariable logistic regression analysis revealed that subjects in the control arm (OR ​= ​11.7, 95% [CI] ​= ​1.3–108, p ​= ​0.03), had a longer duration of surgery, experienced pain and more likely to report unpleasant experience. Adjusting for similar covariates, multivariable linear regression analysis showed that control group patients (β ​= ​8.5 ​mmHg, 95% CI ​= ​6.2–10.8, p ​= ​0.03) had hypertension, experienced fear during surgery and greater fluctuations in the MAP.

**Conclusions:**

A sedative dose of intravenous midazolam during phacoemulsification under topical anesthesia significantly reduces patients’ visual experience, fear and fluctuations in MAP and improves overall surgical experience.

## 1 Introduction

Phacoemulsification is currently the most popular technique for managing cataracts, including dense and mature white cataracts.^1^, ^2^, ^3^The preferred form of anesthesia for this surgery is topical anesthesia with or without conscious sedation.^4^^,^^5^

Patients undergoing cataract surgery, whether using topical or regional anesthesia, experience a variety of visual sensations during the procedure.^6^, ^7^, ^8^, ^9^, ^10^, ^11^ Significantly, some patients have found these intraoperative visual sensations frightening.^12^, ^13^, ^14^, ^15^ Fluctuations in a patient's heart rate (HR), blood pressure (BP), oxygen saturation and other vital parameters can occur during cataract surgery, and these fluctuations may be associated with the patient's visual experience, fear or anxiety during the surgery.^16^ Even cardiac arrest has been reported in an unsedated patient during phacoemulsification under topical anesthesia.^13^

Most ophthalmologists^17^ and anesthesiologists^18^ are aware of cataract patients’ intraoperative visual sensations and their associated psychological effects and have used measures such as preoperative counseling,^19^ music,^15^ and intraoperative sedation to minimize their impact.^5^^,^^20^^,^^21^ Midazolam, a benzodiazepine, is the most commonly used sedative and its high lipid solubility results in a rapid onset of action and clearance. There have been conflicting reports regarding the effectiveness of midazolam in alleviating unpleasant patient experiences during phacoemulsification under topical anesthesia.^4^^,^^22^ A meta-analysis^23^ on topical versus regional anesthesia concluded that there was too much heterogeneity in previously published studies to draw meaningful conclusions regarding the effectiveness of conscious sedation during phacoemulsification.

Hence, we performed this randomized controlled trial to investigate the effect of intravenous midazolam on patients’ intraoperative visual experience, vital parameters, and overall patient experience during phacoemulsification under topical anesthesia.

## 2 Methods

This was a prospective, double-masked, placebo-controlled randomized clinical trial conducted over 20 months at Aravind Eye Hospital, Pondicherry, India. Eligible patients undergoing phacoemulsification with foldable intraocular lens (IOL) implantation under topical anesthesia who met the study criteria (Table 1) were enrolled into the study. The study was approved by the Institutional Review Board and Ethics Committee of the Aravind Eye Care System (Approval number - IRB 201100013). Written informed consent was obtained from each study patient. The study was conducted in accordance with the tenets of the Declaration of Helsinki.

**Table 1:** Inclusion and exclusion criteria.

Inclusion criteria	Exclusion criteria
1 Age range: 45–70 years 2 Eye suitable to undergo surgery under topical anesthesia as per the discretion of the single operating surgeon (RV) 3 Deemed fit to undergo surgery under intravenous midazolam, as per the discretion of a single hospital physician	1. Patient irreversibly blind in fellow eye 2. Complicated cataract, subluxated cataract and poor mydriasis (pupil <5 ​mm size) 3. Coexisting ocular morbidity contributing to decrease in vision such as glaucoma, retinal pathology, corneal opacity and amblyopia 4. Uncontrolled hypertension (SBP ≥160 ​mmHg or DBP ≥100 ​mmHg), or diabetes mellitus (FBS >180 ​mg/dl) 5. Patients at risk of life-threatening side-effects of intravenous sedation such as respiratory depression, cardiac failure, myocardial infarction, anginal pain of <6 months duration, cardiac arrhythmias, and sick-sinus syndrome 6. Patients on β-blockers which might influence cardiac rate and rhythm 7. Patients unable to accurately answer the questionnaire such as dementia, hearing impairment, speech disorder, mental retardation, or patients with any psychiatric ailments

SBP = Systolic blood pressure.

DBP ​= ​Diastolic blood pressure.

FBS = Fasting blood sugar.

### 2.1 Randomization and allocation protocol

Patients were randomized in a 1:1 ratio to receive either intravenous midazolam (midazolam group) or normal saline (control group). The randomization codes were generated using a computer program (random number assignment protocol) and placed in serially numbered sealed opaque envelopes for allocation. The surgeon, counselor, anesthetic nurse recording the vital parameters and patients were masked to the randomization throughout the study.

### 2.2 Pre-operative protocol

One day prior to surgery, all patients underwent a comprehensive ophthalmic evaluation including grading of the cataract density using the lens opacities classification system III.^24^ A single trained counselor then counseled all patients regarding the visual sensations that they may experience during surgery. They were informed that they would be asked about their ‘visual sensations’ and to grade their level of fear and pain subjectively at the end of the surgery. The same counselor, masked to the randomization, then attached an opaque sealed envelope containing the randomization allocation code to each patient's file. The patients were advised to have a light breakfast prior to the surgery.

### 2.3 Intervention

All patients received the following preoperative topical medications in the eye to be operated: tropicamide 1% (Optimide, Microvision, India) (repeated as required), ketolorac tromethamine (Acular LS, Allergan Inc., USA), one drop of preservative-free proparacaine hydrochloride 0.5% (Aurocaine, Aurolab, India) 15, 10, and 5 ​min before surgery and ciprofloxacin eye drops 0.3% (Ciplox, Cipla, India) every 10 ​min for 1 ​h prior to surgery. Two anesthetic nurses were involved in the study. The first anesthetic nurse was masked to the randomization and recorded the systolic BP (SBP), diastolic BP (DBP), HR, and oxygen saturation using the ‘Star 55’ multi-parameter monitor (Larsen and Tubro India Ltd) attached with a pulse-oximeter at four time points: (i) preoperatively 15 ​min prior to surgery, (ii) immediately after corneal incision (intraoperative vital parameters 1), (iii) immediately after IOL implantation (intraoperative vital parameters 2), and (iv) postoperatively four to 6 ​h after the surgery. The second anesthetic nurse opened the sealed randomization envelope and administered intravenously either 0.015 ​mg/kg midazolam or normal saline as per the randomization code 5 ​min before surgery. The patients masked to the intravenous agent used.

A single experienced surgeon (RV), masked to the randomization, performed all the surgeries and refrained from interacting with the patients to maintain the masking process. Electrocardiography (ECG) was continuously monitored by an anesthetist for any adverse changes throughout the perioperative period.

All surgeries were performed with the same constant illumination using a Carl Zeiss OPMI operating microscope with coaxial illumination. Phacoemulsification was done with the Laureate phacoemulsification machine (Alcon Inc., Hunenberg, Switzerland) using a phaco-chop technique through a 2.8 ​mm temporal clear corneal incision, followed by ‘in-the-bag’ acrylic foldable IOL implantation. The fellow eye was covered by a drape throughout the surgery. At the end of each surgery, the surgeon subjectively graded each patient's co-operation during the surgery as ‘excellent’, ‘good’, ‘average’ or ‘poor’. No intracameral anesthetic injection was used during the surgery. Any ocular or systemic complication was recorded.

### 2.4 Post-operative protocol (4–6 ​h after the conclusion of surgery)

**Fig. 1:**
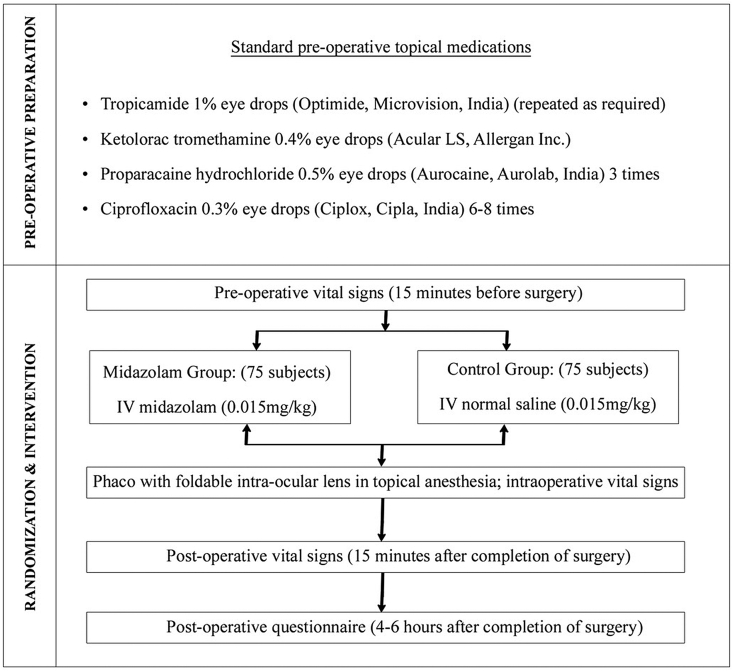
Study flow plan.

The patients were asked to evaluate their ‘visual experience’ on a standardized questionnaire,^12^ which contained questions pertaining to the perception of light, colors, any instrument, hand movement, surgeon/medical staff, sudden increase in vision, no light, or a change in light brightness in the operated eye during surgery. Patients' intraoperative fear (none, mild, moderate or severe), pain (none, mild, significant or unbearable), as well as their overall experience (pleasant or unpleasant) were also recorded in the standardized questionnaire. The study plan (Fig. 1) details the flow of patients through the study.

### 2.5 Outcome measures

Patient's visual experience, fear, pain, fluctuations in mean arterial pressure (MAP), and overall patient experience were the main outcome measures. MAP was calculated at each of the 4 points of blood pressure measurement using the formula: MAP ​= ​DBP + 1/3 (SBP–DBP). Fluctuations in each of the vital parameters of MAP and HR were calculated as follows:

[Intraoperative vital parameter 1 ​+ ​intraoperative vital parameter 2] – [preoperative vital parameter ​+ ​postoperative vital parameter]

### 2.6 Statistical methods

After performing a pilot study and assuming 1:1 randomization, 90% power (α = 0.05), and a precision error of 5% to detect a difference of 20% or more in proportion of patients with unpleasant overall experience between the 2 groups, the required sample size was calculated to be 70 in each group. An additional 5 patients were recruited in each group to give a buffer for patients who might be excluded due to intraoperative complication or who refuse to answer the questionnaire after the surgery.

The data were entered in an Excel datasheet and analyzed using STATA statistical software package (version I/C 12.0, Texas, USA). Continuous variables were presented as mean ​± ​standard deviation and group differences analyzed using student t-test and Mann-Whitney *U* test for non-parametric data. Categorical variables were presented as percentages and group differences analyzed using the Chi square test and Fisher's exact test. Univariate and multiple logistic regression analyses were performed to determine factors influencing unpleasant overall experience and outcomes were expressed as odds ratios (OR) with 95% confidence intervals (CI). Covariates used were drug use (midazolam vs. control), age, gender, hypertension status, other eye lens status, surgical time and pain grade. Fear was not used as a covariate in the multiple logistic regression models as we believe that patients who experienced fear during the surgery would highly likely have reported an unpleasant overall patient experience and thus overshadow other important associations. Using similar covariates, including hypertension status and fear, univariate and multivariable linear regression models were set up to analyze factors associated with wide fluctuations in the MAP. The statistician was however not blinded.

## 3 Results

A total of 150 patients were recruited (75 each in the midazolam group and control group). There was no intraoperative complication, and all patients completed the postoperative questionnaire. The two groups were comparable in terms of demographics and preoperative parameters (Table 2) except that more patients in the midazolam group had a pseudophakic fellow eye than in the control group (p ​= ​0.04). All patients in both groups perceived sensations of light and some colors during surgery (Table 3). The colors seen by patients in both the groups were similar and were mainly white, yellow and blue. Significantly more patients in the control group reported seeing hand movements, surgeon/medical staff and a sudden increase in vision intraoperatively, compared to patients in the midazolam group.

**Table 2:** Comparison of baseline characteristics between groups.

Variable	Midazolam group (n ​= ​75)	Control group (n ​= ​75)	p value
*Age*			
Mean (SD)	60.5(7.0)	59.5(6.7)	0.37∗
Median (Range)	62 (45–70)	60 (45–70)	
*Gender*			
Male (%)	44 (58.7%)	44 (58.7%)	0.99^#^
*Laterality*			
Right eye	41 (54.7%)	45 (60%)	0.509^#^
*LogMAR VA- study eye*			
Mean (SD)	0.45 (0.23)	0.45 (0.25)	0.84^@^
Median (Range)	0.5 (0.2–1.3)	0.3 (0.2–1.3)	
*NS grade (%)*			
NS 1-2	57 (76%)	54 (72%)	0.58^#^
NS 3-4	18 (24%)	21 (28%)	
*Central PSCC (%)*	28 (37.3%)	26 (34.7%)	0.73^#^
*IOP (mm Hg)*			
Mean (SD)	14.73 (1.93)	14.02 (1.89)	0.47∗
Median (Range)	14 (10–20)	13 (10–20)	
*Fellow eye status (%)*			
Phakic	44 (58.7%)	56 (74.7%)	0.04^#^
Pseudophakic	31 (41.3%)	19 (25.3%)	
Aphakic	0 (0%)	0 (0%)	
*Systemic diseases (%)*			
None	34 (45.3%)	35 (46.7%)	0.63^±^
HTN alone	09 (12%)	07 (9.3%)	
DM alone	14 (18.7%)	20 (26.7%)	
HTN ​+ ​DM	10 (13.3%)	06 (8%)	
HTN ​+ ​IHD	04 (5.3%)	05 (6.7%)	
HTN ​+ ​DM ​+ ​IHD	03 (4%)	00 (0%)	
Others	00 (0%)	01 (1.3%)	
HTN ​+ ​DM ​+ ​Others	01 (1.3%)	01 (1.3%)	

SD = Standard deviation; LogMAR VA ​= ​Logarithm of Minimum Angle of Resolution visual acuity.

NS = Nuclear sclerosis; PSCC = Posterior subcapsular cataract; IOP = Intra-ocular pressure.

HTN = Hypertension; DM ​= ​Diabetes mellitus; IHD = Ischemic heart disease.

∗*t*-test; ^#^Chi-square test; ^@^Mann-Whitney *U* test; ^±^Fisher's exact test.

**Table 3:** Comparison of visual sensations between groups.

Type of visual sensation		Midazolam group, n (%)	Control group, n (%)	p value
Presence of light		75 (100%)	75 (100%)	NA
Presence of color		75 (100%)	75 (100%)	NA
Specific color	White	56 (74.7%)	53 (70.7%)	0.71
	Yellow	48 (64%)	50 (66.7%)	0.86
	Red	15 (20%)	24 (32%)	0.14
	Blue	32 (42.7%)	33 (44%)	0.99
	Green	9 (12%)	17 (22.7%)	0.13
	Rainbow	3 (4%)	3 (4%)	0.99
	Not sure	2 (2.7%)	1 (1.3%)	0.99
Sudden flash of light at any point during surgery		23 (30.7%)	34 (45.3%)	0.09
Any instrument		11 (14.7%)	20 (26.7%)	0.11
Hand movement		10 (13.3%)	27 (36%)	<0.01
Surgeon/medical staff		1 (1.3%)	8 (10.7%)	0.04
Sudden increase in vision during surgery		9 (12%)	24 (32%)	<0.01
No light at any point in surgery		0 (0%)	3 (4%)	0.24
Change in brightness during surgery		3 (4%)	9 (12%)	0.13

A greater number of patients in the control group experienced fear (p ​< ​0.001) and an unpleasant overall surgical experience (p ​< ​0.001) compared to those in the midazolam group (Table 4). Additionally, 6 patients (8%) in the control group versus none in the midazolam group had moderate to severe pain although this difference was not statistically significant (p ​= ​0.06).

**Table 4:** Fear, pain, overall surgical experience, fluctuations (Δ) in MAP and HR, duration of surgery and surgeon's score on patient cooperation.

Variable	Midazolam group	Control group	p value
*Fear (%)*			
None	52 (69.3%)	20 (26.7%)	<0.001
Mild	21 (28%)	39 (52%)	
Moderate	2 (2.7%)	15 (20%)	
Severe	0	1 (1.3%)	
*Pain (%)*			
None	39 (52%)	32 (42.7%)	0.06
Mild	36 (48%)	37 (49.3%)	
Significant	0 (0%)	5 (6.7%)	
Unbearable	0 (0%)	1 (1.3%)	
*Unpleasant overall surgical experience*	1 (1.3%)	16 (20.3%)	<0.001
Δ MAP	7.2 ​± ​5.3	16.9 ​± ​7.9	<0.001
Δ MAP in HTN (n ​= ​46)	8.3 ​± ​4.8	23.1 ​± ​7.9	<0.001
Δ MAP in non HTN (n ​= ​104)	6.6 ​± ​5.7	14.9 ​± ​6.9	<0.001
Δ HR	4.60 ​± ​6.8	12.93 ​± ​11.4	<0.001
*Duration of surgery (minutes)*			
Mean (Range)	5.97 (4–8)	6.24 (4–9)	0.10
Median (SD)	6 (0.94)	6 (1.05)	
*Surgeon's score on patient cooperation*			
Excellent	50 (66.7%)	35 (46.7%)	0.10
Good	21 (28%)	33 (44%)	
Moderate	03 (4%)	04 (5.3%)	
Poor	01 (1.3%)	03 (4%)	

MAP ​= ​Mean arterial pressure, HTN = Hypertension, HR= Heart rate.

**Fig. 2:**
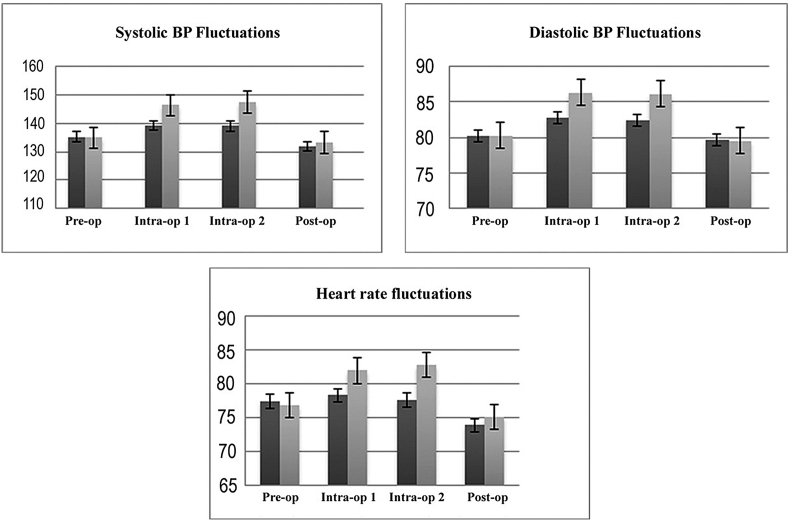
Comparison of fluctuations in blood pressure and heart rate between study and control patients at different time points during the study.

Patients in the control group experienced greater fluctuation in MAP and HR compared to those in the midazolam group (Table 4). Within the control group, hypertensive patients experienced significantly greater fluctuations in MAP (ΔMAP ​= ​23.1 ​± ​7.9 ​mmHg) compared to normotensive patients (ΔMAP ​= ​14.9 ​± ​6.9) (p ​= ​0.0003). However, the difference in fluctuations in MAP between hypertensive (ΔMAP ​= ​8.3 ​± ​4.8) and normotensive patients (ΔMAP ​= ​6.6 ​± ​5.7) was not statistically significant in the midazolam group (p ​= ​0.11). Fig. 2 shows a graphic representation of fluctuations in vital parameters at four time points during the study. There were no significant differences in the duration of surgery and surgeon's score for patient cooperation between the two groups (Table 4). There was a significant difference in the ΔMAP between those with pleasant (10.8 ​± ​7.7 ​mmHg) vs. unpleasant (22.2 ​± ​5.3 ​mmHg) overall surgical experience (p ​< ​0.001, Mann-Whitney).

Adjusting for age, gender, hypertension status and fellow eye lens status, univariate and multivariable logistic regression analysis revealed that patients in the control group were nearly 12 times more likely to report an overall unpleasant surgical experience (OR ​= ​11.7, 95% CI ​= ​1.3–108, p ​= ​0.03) compared to those in the midazolam group (Table 5). Additionally, increase in the duration of surgery (OR ​= ​2.4, 95% CI ​= ​1.1 to 5.1 for every 1-min increment in duration, p ​= ​0.025), and greater pain (OR ​= ​4.6, 95% CI ​= ​1.5 to 14.0, p ​= ​0.007) were also significantly associated with an overall unpleasant surgical experience. Adjusting for similar covariates, univariate and multivariable linear regression analysis showed that patients in the control group (8.5 ​mmHg greater increment in MAP in control group vs. midazolam group, p ​= ​0.001), hypertensive patients (4.2 ​mmHg greater increment in MAP in hypertensives vs. normotensives) and those who experienced fear during surgery (2.3 ​mmHg greater increment in MAP in those experiencing fear vs. those with no fear) had significantly greater fluctuation in MAP (Table 5).

**Table 5:** Univariate and multivariable analysis of factors predicting an overall unpleasant experience (logistic regression) and fluctuations in mean arterial pressure (Linear regression).

Variable	Factors	Logistic regression (OR, 95%CI)^a^		Linear regression (β, 95%CI)^b^	
		Univariate	Multivariable	Univariate	Multivariable
Sedation	Control group vs. midazolam group	**20.1 (2.6-155)∗**	**11.7 (1.3-108)∗**	**9.74 (7.5-11.9)∗**	**8.5 (6.2–10.8)∗**
Age	10-year increment	1.29 (0.6**–**2.8)	1.4 (0.5**–**3.9)	-1.24 ( -3.2**–**0.7)	-0.83 (-2.4–0.7)
Gender	Female vs. male	0.77 (0.3**–**2.1)	0.96 (0.3**–**3.6)	-0.47 (-3.2**–**2.3)	0.1 (-1.9–2.2)
BCVA^c^	0.1 LogMAR increment	0.18 (0.01**–**2.8)	---	0.33 (-5.3–6.0)	---
Blood pressure	Hypertensive vs. normotensive	1.27 (0.4**–**3.6)	1.84 (0.4**–**7.7)	**3.3 (0.4–6.2)∗**	**4.2 (1.9–6.4)∗**
Fellow eye status	Phakic vs. pseudophakic	1.22 (0.4**–**3.7)	2.1 (0.5**–**9.9)	1.7 (-1.1–4.6)	---
Surgical time	1-minute increment	0.71 (0.4**–**1.2)	**2.4 (1.1–5.1)∗**	0.36 (-0.9–1.7)	-0.32 (-1.4–0.7)
Intraoperative fear	Fear present vs. fear absent	**65.7 (13.7–313)∗**	--	**5.5 (3.7–7.2)∗**	**2.3 (0.6–4.0)∗**
Intraoperative pain	Pain present vs. pain absent	**5.6 (2.1–15.2)∗**	**4.6 (1.5–14)∗**	**3.2 (0.9–5.4)∗**	1.5 (-0.3–3.3)

∗p<0.05.

a Logistic regression for factors predicting unpleasant overall experience.

b Linear regression for factors predicting greater fluctuation in mean arterial pressure, Δ Mean arterial pressure (R^2^=0.46).

c Baseline best corrected visual acuity (pre-operative).

## 4 Discussion

We found that fewer patients who had sedation with intravenous midazolam during routine phacoemulsification and IOL implantation under topical anesthesia perceived hand movements, surgeon/medical staff and a sudden increase in vision during the surgery compared to patients who were not sedated. Those with sedation also showed lesser fluctuations in vital parameters compared to those without sedation, especially in patients with hypertension. Those with sedation were also more likely to report a pleasant overall patient experience compared to those without sedation.

Our patients were advised to consume a light meal before the surgery. This in accordance with the suggestion by Seet et al.^25^ Prolonged fasting may affect patients' psychological and physical well-being and may lead to dehydration, hypoglycemia and irritability.^26^ Our findings are supported by Venkatakrishnan et al^20^ who found that 92% of cataract patients given intravenous midazolam reported a pleasant overall experience compared to 77% of patients without sedation. Similarly, Dogan et al^27^ compared two different sedatives and reported greater patient and surgeon satisfaction and lower pain scores using a combination of midazolam and fentanyl compared to dexmedetomidine. Fernandes et al^28^ reported favorable results and excellent patient satisfaction from a large cohort of 106 patients using a combination of topical and intracameral anesthesia along with conscious sedation but there was no control group. Habib et al^22^ however, found no difference in the level of patient satisfaction, pain and anxiety scores in patients who received midazolam compared to controls. Their study differed from ours in that their patients also received additional intracameral lidocaine, thus possibly further reducing pain and positively influencing the overall patient experience. In addition, all their patients were counseled immediately prior to surgery, which probably helped in alleviating patients’ anxiety. This, along with the intracameral lidocaine, could have masked the influence of conscious sedation in their study.

Using multivariable regression models, we found that patients who did not receive midazolam were nearly 12 times more likely to report an unpleasant patient experience than those who did. We also found that a longer duration of surgery and presence of intraoperative pain were associated with an overall unpleasant patient experience. These findings suggest that, besides sedation, minimizing the duration of surgery and reducing intraoperative pain (e.g., by using additional intracameral lidocaine) may improve the overall patient experience. To the best of our knowledge, we believe that ours is the first paper to study the influence of various factors on overall patient experience during phacoemulsification under topical anesthesia.

We also studied the influence of conscious sedation on vital parameters such as BP and HR. We did not exclude those with hypertension and ischemic heart disease in an attempt to study the influence of sedation in this subgroup of patients. Yap et al.^29^ reported a significant elevation in both SBP and DBP during phacoemulsification under topical anesthesia, a result similar to ours. The authors also found that hypertensive patients did not have an increased risk of BP rise intraoperatively. However, this study had a relatively small sample size (n ​= ​46), thus making it underpowered for evaluating risk factors associated with BP fluctuations. Dogan et al.^27^ demonstrated hemodynamic stability in all their patients who were administered 2 different sedatives, similar to what we have demonstrated in the midazolam group. Zhao et al.^23^ performed a meta-analysis of randomized controlled trials comparing topical and regional anesthesia and reported that topical anesthesia was not a suitable choice for patients with higher initial BP. However, the authors conceded that there is insufficient evidence regarding the role of conscious sedation in phacoemulsification using topical anesthesia and hoped that there will be more studies in the future investigating this area.

We found that the fluctuations in BP were significantly attenuated with intravenous midazolam. We also found that patients with hypertension demonstrated greater fluctuations in BP, even after adjusting for sedation, though hypertensive patients in the midazolam group showed much lesser fluctuations than controls. Not surprisingly, we also found intraoperative fear to be a risk factor for greater BP fluctuations. Similarly, Moreno-Montañés et al.^30^ showed that hypertension is the most important risk factor for anesthesiologic interventions during phacoemulsification under topical anesthesia and very high intraoperative BP was the commonest cause for anesthesiologic intervention. Such sudden rise in BP may be detrimental in patients and may rarely precipitate life-threatening cardiovascular events. The authors presented an excellent Poisson regression model but failed to evaluate the influence of conscious sedation as all 1010 patients in their study also received oral bromezapem 2 ​h before surgery. We have used MAP that takes both SBP and DBP into account and we have measured BP at 2 different time points during surgery. We believe that ours is the first study to report on the factors influencing fluctuations in BP during phacoemulsification under topical anesthesia. Our study suggests it may be prudent to sedate patients, especially those with hypertension, to prevent intraoperative BP spikes. Alleviating intraoperative fear through such measures as adequate preoperative counseling and having a relaxed environment during surgery may also help prevent excessive BP fluctuations during surgery, though these interventions require further study.

There was no statistically significant difference in the surgeon's rating of patient co-operation between the two groups, although almost two-thirds of those under conscious sedation showed excellent co-operation compared to only half of the controls. A previous cataract surgery under topical anesthesia in the fellow eye did not seem to influence patients' overall experience during surgery. We find this surprising as one would imagine that patients undergoing their second cataract surgery would be better prepared mentally to face the second surgery. Interestingly, Ursea et al.^31^ have shown that the majority of patients experience more pain during the second phacoemulsification surgery under topical anesthesia compared to the first.

We believe that it is important to prevent excessive sedation during surgery and hence a dose of midazolam that causes only conscious sedation is recommended. Excessive somnolence may cause rhythmic head movement and prevent patients from making subtle eye movements as occasionally requested by the surgeon. We also found no serious adverse events attributable to the use of midazolam suggesting that it is safe to use even in patients with systemic risk factors like hypertension, ischemic heart disease, and diabetes.

The strengths of our study are the use of a previously described, standardized questionnaire, recording vital parameters twice at specific stages during the surgery, recording both subjective patient experience and objective vital parameters and the randomization and masking protocols adopted. The drawbacks are the exclusion of patients on b-blockers and those with other systemic ailments such as arrhythmias. Also, we did not study the influence of other socio-demographic factors such as education and personality type on patient experience. Compared to western cohorts, this study incorporated a simpler scale for subjective grading of fear and pain, as the relatively lower literacy rate of our patients prevented us from using complex but more informative scales. The post-operative questionnaire was conducted 4–6 ​h after surgery, since the duration of action of midazolam is usually 4–6 ​h. However, in few patients its effect can last up-to 1 day. But conducting the questionnaire the next day might not be feasible since cataract surgery is usually a day care procedure, most patients would be discharged the same day. Also, an overnight rest might have an influence on the anxiety and overall experience of the patient.

## 5 Conclusions

We found that conscious sedation using intravenous midazolam was associated with reduced perception of visual sensations, fluctuations in MAP, fear and a pleasant overall experience during topical phacoemulsification under topical anesthesia.

## Study Approval

The study was approved by the Institutional Review Board and Ethics Committee of the Aravind Eye Care System (Approval number - IRB 201100013).

## Author Contributions

The authors confirm contribution to the paper as follow: RV - Conceptualization, Methodology, Investigation, Supervision, Conducting the study.

HK - Data curation, Writing- Original draft preparation, Investigation, Conducting the study.

SS - Writing- Reviewing and Editing.

MG - Writing- Reviewing and Editing.

KGAE - Conceptualization, Methodology, Writing- Reviewing and Editing, Supervision.

All authors reviewed the results and approved the final version of the manuscript.

## Acknowledgements

Thanks to all the peer reviewers for their opinions and suggestions.

## Funding

This research did not receive any specific grant from funding agencies in the public, commercial, or not-for-profit sectors.

## Conflict of Interest

The authors declare that they have no known competing financial interests or personal relationships that could have appeared to influence the work reported in this paper.

## Abbreviations

MAP: Mean Arterial Pressure
HR: Heart Rate
BP: Blood Pressure
IOL: Intra-Ocular Lens
IRB: Institutional Review Board
SBP: Systolic Blood Pressure
DBP: Diastolic Blood Pressure
ECG: Electrocardiography
OR: Odds Ratio
CI: Confidence Interval
SD: Standard Deviation
LogMAR VA: Minimum Angle of Resolution Visual Acuity
NS: Nuclear Sclerosis
PSCC: Posterior Subcapsular Cataract
IOP: Intra-Ocular Pressure.
HTN: Hypertension
DM: Diabetes Mellitus
IHD: Ischemic Heart Disease
